# Conditional generative models enable targeted exploration of MAX phase design space

**DOI:** 10.1039/d6tc01390b

**Published:** 2026-07-23

**Authors:** Jamie Swaine, Cyprien Bone, Prakriti Kayastha, Matthew T. Darby, Ewan Galloway, Keith T. Butler

**Affiliations:** a Department of Chemistry, University College London, Kathleen Lonsdale Building, Gower Pl London WC1E 6BS UK k.t.butler@ucl.ac.uk; b AWE Aldermaston, Reading Berkshire RG7 4PR UK

## Abstract

MAX phases (M_*n*+1_AX_*n*_), precursors to MXenes, span a vast compositional space, motivating efficient computational screening for synthesisable candidates. We employ CrystaLLM−π, a large language model fine-tuned on 6179 double transition-metal MAX phases, and demonstrate its ability to generate novel structures consistent with known experimental trends. Using a conditioning vector with two dimensions (a statistically derived MXene derivative count and a surrogate for A-site binding energy), the model was able to target MXene-favourable regions of phase space for generation. Specific condition vectors double novel stable structure generation rates *versus* unconditioned baselines. Of ten compositionally novel candidates, five exhibit DFT-validated (*meta*)stability (*E*_hull_ < 0.050 eV per atom). This work showcases the potential for autoregressive generative models to explore targeted materials' spaces, offering a scalable framework for accelerated discovery in compositionally complex systems.

## Abstract 2

MAX phases (M_*n*+1_AX_*n*_), precursors to MXenes, span a vast compositional space, motivating efficient computational screening for synthesisable candidates.

## Graphical abstract

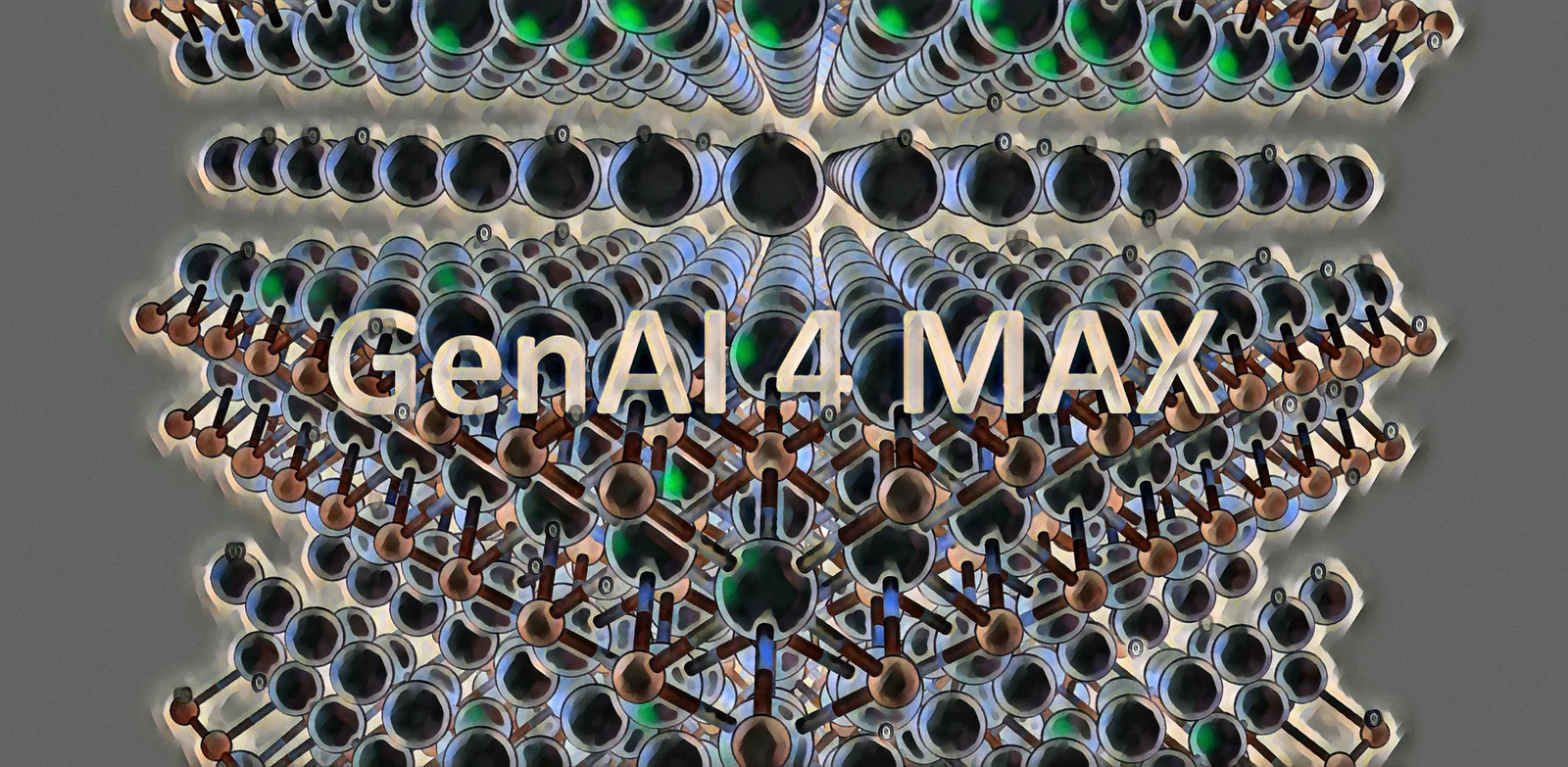

## 1. Introduction

MAX phases constitute a class of nano-laminated materials that combine ceramic-like robustness with metallic conductivity. They have found use in many applications, including corrosion- and radiation-resistant coatings for nuclear materials,^1^ energy storage,^2^ and catalysis.^3^ Represented by the general formula M_*n*+1_AX_*n*_, MAX phases comprise an early transition metal M-site, an A-site which is generally a group 13–14 element, and a non-metal X-site, which is most often carbon, nitrogen, or boron. Scientific interest in these phases has increased rapidly in recent years, with 342 experimentally synthesised compositions reported as of 2024, approximately half of which were discovered after 2018. Notably, MAX phases also serve as precursors to MXenes, two-dimensional derivatives that retain many of the parent MAX characteristics while offering tunable surface chemistries. Represented by the general formula M_*n*+1_X_*n*_T_*x*_, MXenes arise from the selective removal of the A-site element and the subsequent formation of surface terminations, T, whose identity depends on the synthetic and post-processing environments.

As of 2024, 28 M-site elements, 28 A-site elements, and 6 possible X-site elements have been reported in experimentally synthesised MAX phases.^4^ Even under a single stoichiometric family the naïve expansion of the element sets leads to more than 4700 theoretical ternary compounds before accounting for variation in the crystal structure itself. Consequently, it is both efficient and necessary to identify and evaluate theoretically stable candidates *via* computational methods, thereby refining and guiding experimental search.

The application of machine learning techniques in chemistry has increased sharply over the past decade, with such methods now frequently integrated with traditional theoretical and computational approaches to alleviate the computational bottleneck associated with *ab initio* calculations.^5–9^ Deep neural networks have demonstrated accuracy comparable to that of density functional theory (DFT) while performing evaluations orders of magnitude faster.^10,11^ In particular generative models offer the promise of predicting new viable materials, with desired properties, on demand.^12^

Recently there have been a number of studies reporting transformer-based “large language models” (LLMs) adapted for materials design.^13–15^ These approaches leverage the flexibility and scalability of text-based transformers for modelling complex chemical space.^16–19^

Beyond transformer-based approaches, a range of contemporary frameworks for crystal structure generation have emerged. Physics-guided deep learning models that incorporate explicit symmetry and stability constraints have been of particular interest.^20–30^ These models offer stronger physical grounding and reduce the need for extensive post-processing. Together, generative and physics informed approaches demonstrate a growing shift toward computational strategies that tightly couple machine learning with underlying materials chemistry and physics.

In this study, we consider the application of CrystaLLM,^13^ a transformer-based autoregressive generative model, trained on crystallographic information files (CIFs) which encode lattice parameters, space-group, and atomic species. This model was recently updated to include conditioning mechanisms as of 2025 allowing for property guided generation.^31^ This updated conditioning model is subsequently referred to as CrystaLLM−π. Under conditional generation this model produces crystal structures in the same way as the base model, but with the additional objective of satisfying user specified targets, encoded in the condition vector. Here we condition CrystaLLM−π to explore specific regions of MAX phase design space.

A secondary investigation was also conducted on MAB phases, an under-explored subclass of MAX phases that have recently attracted attention for similar applications to those described previously, but possess boron in the X site. This represented a low-shot learning regime, within which CrystaLLM−π successfully generated novel structural motifs that were likewise validated through DFT calculations at the PBE level.

As part of these investigations, we utilise the GNNs (graph neural networks), MACE (message passing atomic cluster expansion) and ALIGNN (atomistic line graph neural network) for energy and property predictions. MACE and ALIGNN are members of a class of models known as MLIPs (machine learned inter atomic potentials), which have been shown to offer accuracy on the order of DFT calculations at a cost similar to deriving classical interatomic potentials.^32^

The results of this study demonstrate how the capabilities of CrystaLLM−π can be leveraged for targeted exploration of MAX phase chemistry. Across tens of thousands of structural generations, we show that model conditioning not only promotes broader exploration of the compositional and structural design space, but also yields crystal structures that exhibit strong fidelity to their imposed conditioning. Furthermore, CrystaLLM−π was shown to generate structures with out-of-sample chemical composition that were also thermodynamically (*meta*)stable, under DFT at the PBE level of theory. The results highlight the utility of conditioned autoregressive transformer-based models for materials design and discovery.

## 2. Results and discussion

In this section some technical terms are used, which are defined in detail later in the methodology, but which we provide a brief definition of here, to aid with readability. Novelty of structures is defined by comparing the composition and the structure of the generated material against all of the data used to train the models, if no matches are found, the material is considered novel, full details are in Section 4.7. Metastability is defined as the structure having an energy above the convex hull less than 100 meV per atom, metastability is generally calculated using MACE formation energy, but is later validated with DFT formation energies, full details are in Section 4.3. Conditioning mechanisms are the means by which the generative process is guided towards desired properties (the conditioning properties), two types PKV_prefix_ and PKV_residual_ are employed, the details are given in Section 4.4.

Two conditioning properties were employed in this study. The first served as a preliminary screening step, assessing structures based on their potential MXene derivative count. The second condition involved a sublattice perturbation of A-site elements, used as a surrogate for vacancy formation energy and free-energy evaluation.

A MAX phase was designated as having a derivative by cross-referencing the fine-tuning dataset with the MXene dataset from aNANt, which are described in Section 4.2. Two key assumptions were made in calculating the derivative count for any given species. First, the stoichiometry of the parent compound was not assumed to be preserved during etching.^33^ Second, the nature of MXene terminations was assumed to be dictated entirely by the etching and post-processing environment, and thus fully customisable.^34^ On this basis, a MXene derivative was defined as any subset of the transition-metal elements comprising the parent MAX phase.

Based on these assumptions, 6013 structures from the fine-tuning dataset (not including the MAX/MAB-like structures) were identified as possessing between 1 and 172 potential MXene derivatives. This condition vector is entirely statistically derived and simply serves to guide generation with respect to elemental contents of the MAX phase. This count was normalised using a min–max scaler between 0 and 1, meaning that a value of 0.00 would aim to push generation towards a space associated with the lowest number of derivatives, where a value of 1.00 would suggest the greatest derivative count.

To derive a surrogate for the binding energy of the A-site, and thus obtain a meaningful representation of vacancy formation and free-energy values, a perturbation-evaluation procedure was applied to the A-sublattice. The A-site layer was displaced by 1% of the *c* lattice parameter in either direction, and for each perturbation the *E*_hull_ metric was computed using MACE, see Section 4.3.

**Fig. 1:**
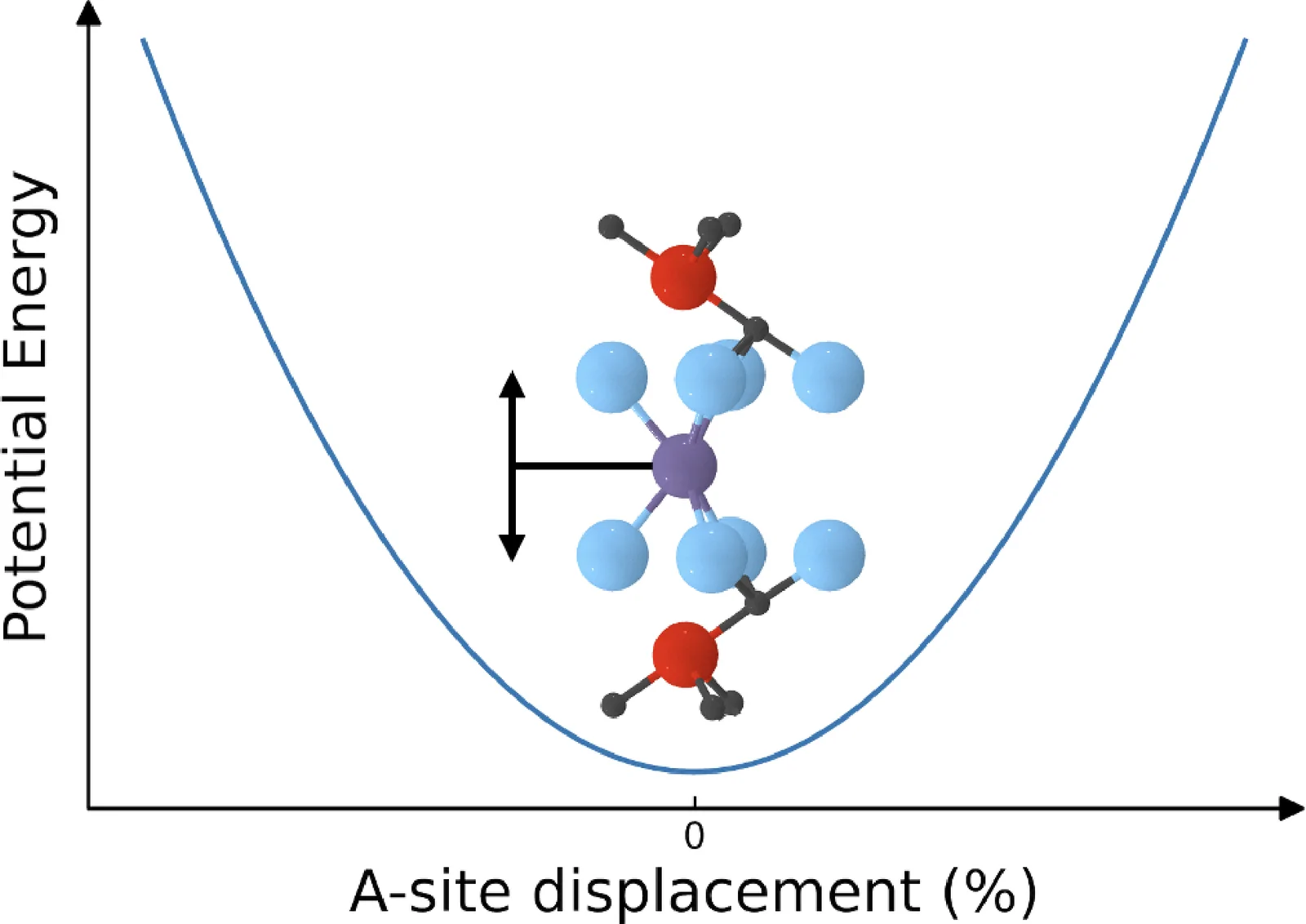
Schematic of an A-site sublattice perturbation in a representative double transition metal MAX phase unit cell and the corresponding potential energy change. The A-site atom is shown in purple, transition metal M-site atoms in light blue and red, and X-site atoms in black.

The curvature of the potential well was estimated using a Taylor series expansion about the equilibrium geometry, with the unperturbed structure taken as the zeroth-order reference point. A schematic illustration of this procedure is shown in Fig. 1. Assuming that the energy above hull is minimized at *E*(0), higher-order terms can be neglected, giving:1
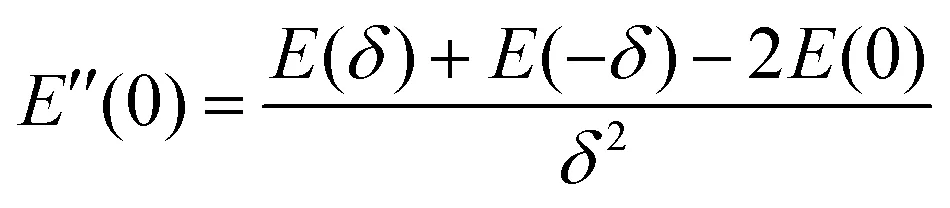


**Fig. 2:**
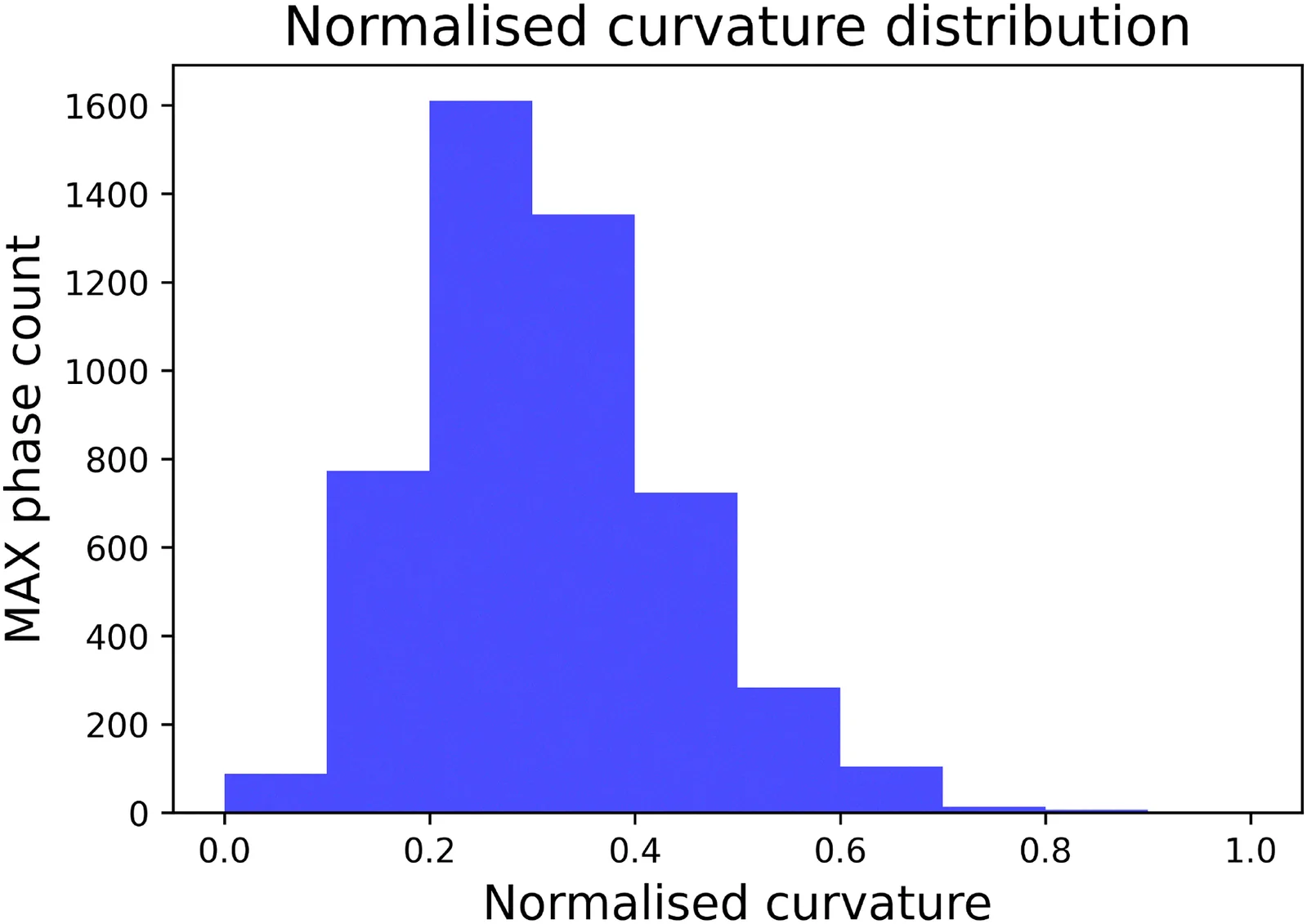
Histogram displaying the min–max normalised A-site well curvature values against MAX phase count within the training dataset.

This was then normalised across the dataset using min–max scaling to give a value between 0 and 1 as was the case in the previously considered potential MXene derivative count dimension. A histogram displaying this distribution can be seen in Fig. 2.

Further justification for this method was provided by the observation that, in all cases, the mapped potential energy surface was convex. Moreover, taking the noise level of MACE to be approximately 1–5 meV per atom,^5^ the signal exceeded the noise in every instance when considering the upper bound of 5 meV per atom. Additionally, a harmonic regime was preserved in over 80% of cases, supporting the conclusion that this procedure reliably probed the bottom of the potential energy well.

### 2.1. Conditional generation

We assess the impact of conditional prompting on the properties of the generated structures. In this experiment we do not specify the composition of the prompt, but only use the conditioning vector to guide generation, we term this non-compositional prompting. Directly evaluating the effect of the first conditioning property, the MXene derivative count, was challenging, as this value is purely statistical and would require knowledge of the complete set of possible MXene derivatives at the point of generation to assess fidelity. By contrast, the second conditioing property, A-site well curvature, can be evaluated more directly.

**Fig. 3:**
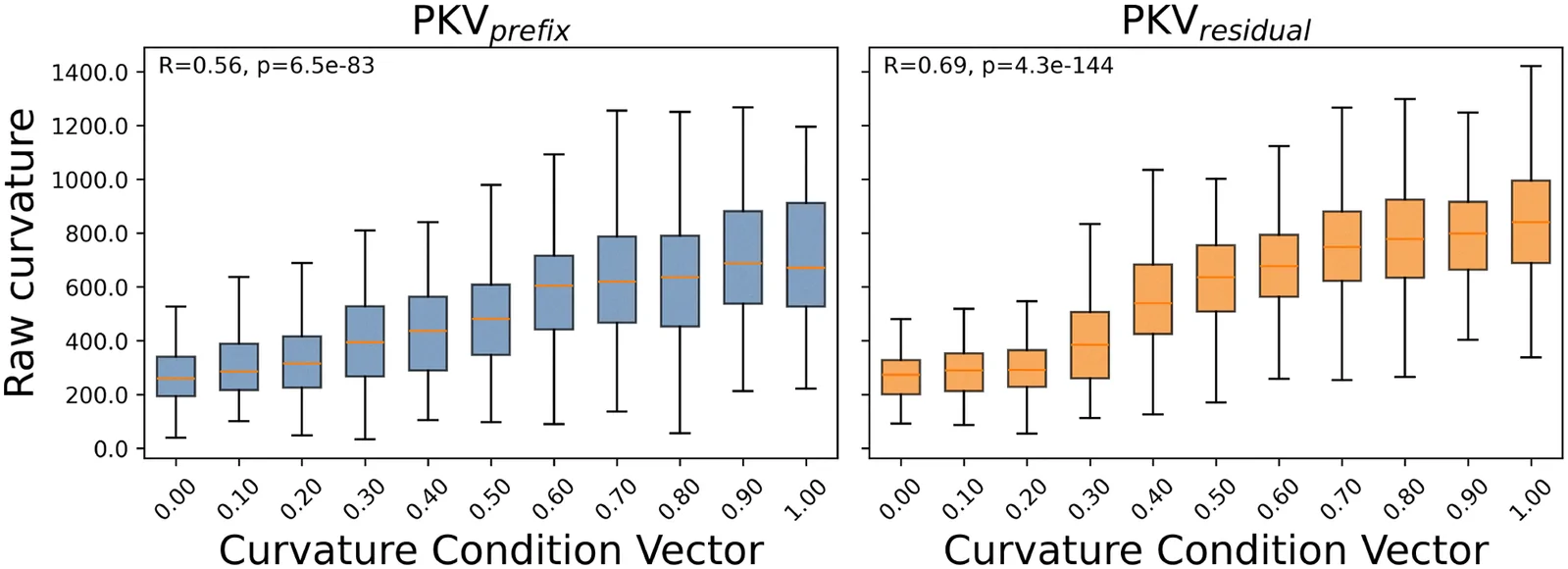
Plots showing the calculated curvature (arbitrary units) across a sweeping A-site well condition vector, with constant MXene derivative count dimension.

To this end, we consider the 1000 structures generated under non-compositional prompting (see Section 4.6.1) for each of the two conditioning mechanisms PKV_prefix_ and PKV_residual_ models (see Section 4.4), using a fixed MXene derivative count condition of 0.95 while sweeping the A-site well curvature from 0.00 to 1.00 in increments of 0.10. The resulting A-site well curvatures were calculated as described in the methods and plotted against the condition vector values. The results are shown in Fig. 3.

We observe a clear upward trend in A-site curvature with increasing values of the A-site condition vector. For PKV_prefix_, the Pearson correlation is *r* = 0.56 (*p* = 6.5 × 10^−83^), while for PKV_residual_ it is *r* = 0.69 (*p* = 4.3 × 10^−144^), detailing very high significance.

The disparity in *r* values can be understood by considering the way missing values are handled by each model throughout the dataset. The imputation required to handle some structures in the PKV_prefix_ model may be detracting from physically meaningful properties associated with some structures and thus diluting the meaning of the condition vector itself.

Before considering rates of novel (*meta*)stable structure generation, we first assess the extent to which non-compositional prompting yields valid MAX phase compositions and structures, independent of stability or novelty. A structure was classified as a valid MAX phase if it satisfied the (M,M′)_*n*+1_AX_*n*_ stoichiometry (Section 4.2) and crystallised in one of the space groups associated with the MAX phase structure type: *P*6_3_/*mmc*, *R*3̄*m*, *P*3̄*m*1, *P*6̄*m*2, *Cmcm*, and *P*6_3_*mc*. Across all 59 400 non-compositional generations (three model variants × 19 800 structures each), ∼99% were valid MAX phases. This confirms that non-compositional prompting reliably yields structurally and compositionally valid MAX phase candidates, and that the novel (*meta*)stable structure generation rates discussed below are therefore computed relative to a near-fully valid generated pool.

**Fig. 4:**
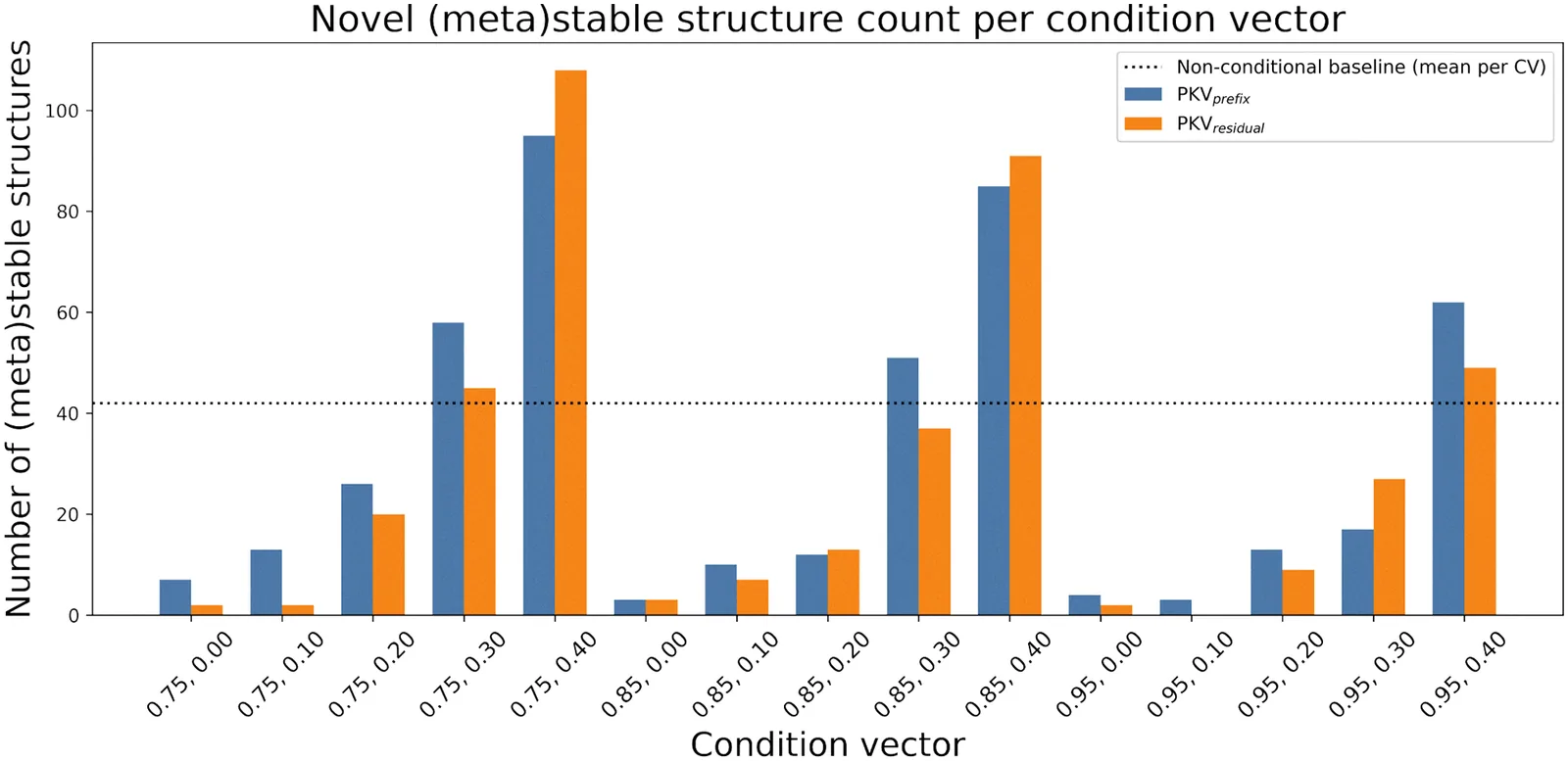
Bar chart detailing novel stable structure generation counts with respect to condition vector under non-compositional prompting. The dashed line indicates the non-conditional model baseline. Prompting performed within a condition vector space biased toward high potential MXene derivative counts and low A-site well curvature.

We now concentrate on generation in the region corresponding to high MXene derivative counts and low A-site well curvature. This region was defined as 0.75–0.95 (inclusive, step size 0.10) for the MXene derivative dimension and 0.0–0.40 (inclusive, step size 0.10) for the A-site well curvature dimension. Within this defined sweep space, biased toward high MXene derivative counts and weak A-site binding, non-compositional prompting showed that the PKV_prefix_ and PKV_residual_ models generated 459 and 415 novel metastable structures,^35^ respectively, compared to 644 from the baseline model. These results correspond to novel structure generation rates of 2.32%, 2.10%, and 3.25% for PKV_prefix_, PKV_residual_, and the baseline, respectively. It is evident, however, that relative to the non-conditional baseline, performance varies considerably across condition vectors. Some vectors outperform the baseline by a wide margin, while others under-perform. This variability is clearly illustrated in Fig. 4.

At (0.75, 0.40) and (0.85, 0.40), both models significantly outperformed the baseline, with odds ratios of 2.09–2.71 (*p* < 0.001), indicating that conditioning under these settings more than doubled the likelihood of generating metastable structures. By contrast, at (0.75, 0.30), neither PKV_prefix_ nor PKV_residual_ achieved statistical significance (95% CI, *p* < 0.05). At (0.95, 0.40), PKV_prefix_ showed marginal significance (OR = 1.50, *p* = 0.028) while PKV_residual_ did not. From these results we can conclude that generation rates are highly dependent on the specific condition vector employed.

Overall we can clearly see that conditioning not only has a meaningful impact on specific structural features but at specific condition vectors it is encouraging the model to explore a space capable of yielding more structurally novel and stable compounds than its non-conditioned counterpart. It can also be observed that novel stable structure generation rates increase with rising A-site well curvature, a trend that aligns with chemical intuition, *i.e.* a more tightly bonded A-site is more likely to yield a relatively more stable structure. This highlights the delicate balance that needs to be achieved to propose stable, but easily exfoliated structures. The conditioning vector sweep was not extended beyond this range, as the purpose of the experiment was to focus on a region that was more likely to yield “MXeneable” structures, not to generate arbitrary novel structures.

### 2.2. Elemental prevalence

Having established that conditioning can steer structural features and improve rates of novel structure generation, we next examine the elemental distribution across the previously defined set of novel metastable structures, the definition of novelty is explained in Section 4.7. This set has already been filtered for metastability (*E*_hull_ < 0.1 eV per atom) and stoichiometric compliance (M_*n*+1_AX_*n*_), the latter confirming that the generated structure satisfies the (M,M′)_*n*+1_AX_*n*_ formula for single or double transition-metal MAX phases using elements drawn from the sets described in Section 4.2 and crystallising in one of the associated space groups (see Section 4.1). Both checks are applied at step C of the generation and filtering pipeline (Fig. 6), therefore the analysis that follows concerns relative thermodynamic favourability and elemental generation frequency within this metastable set.

**Fig. 5:**
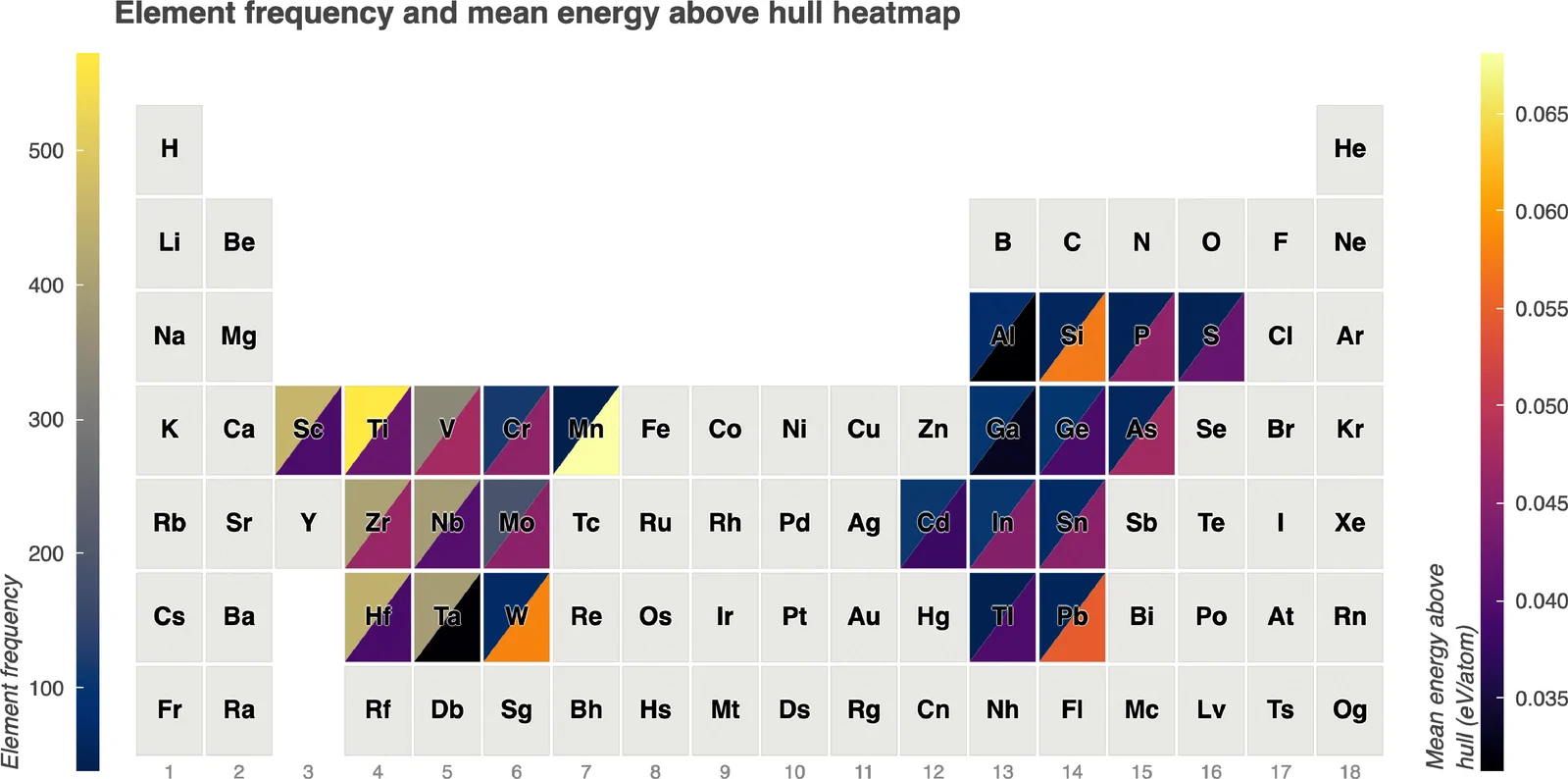
Element frequency heatmap for the set of novel stable structures showing element usage weighted by stoichiometry (upper left corner) and the mean energy above hull (lower right corner).

**Fig. 6:**
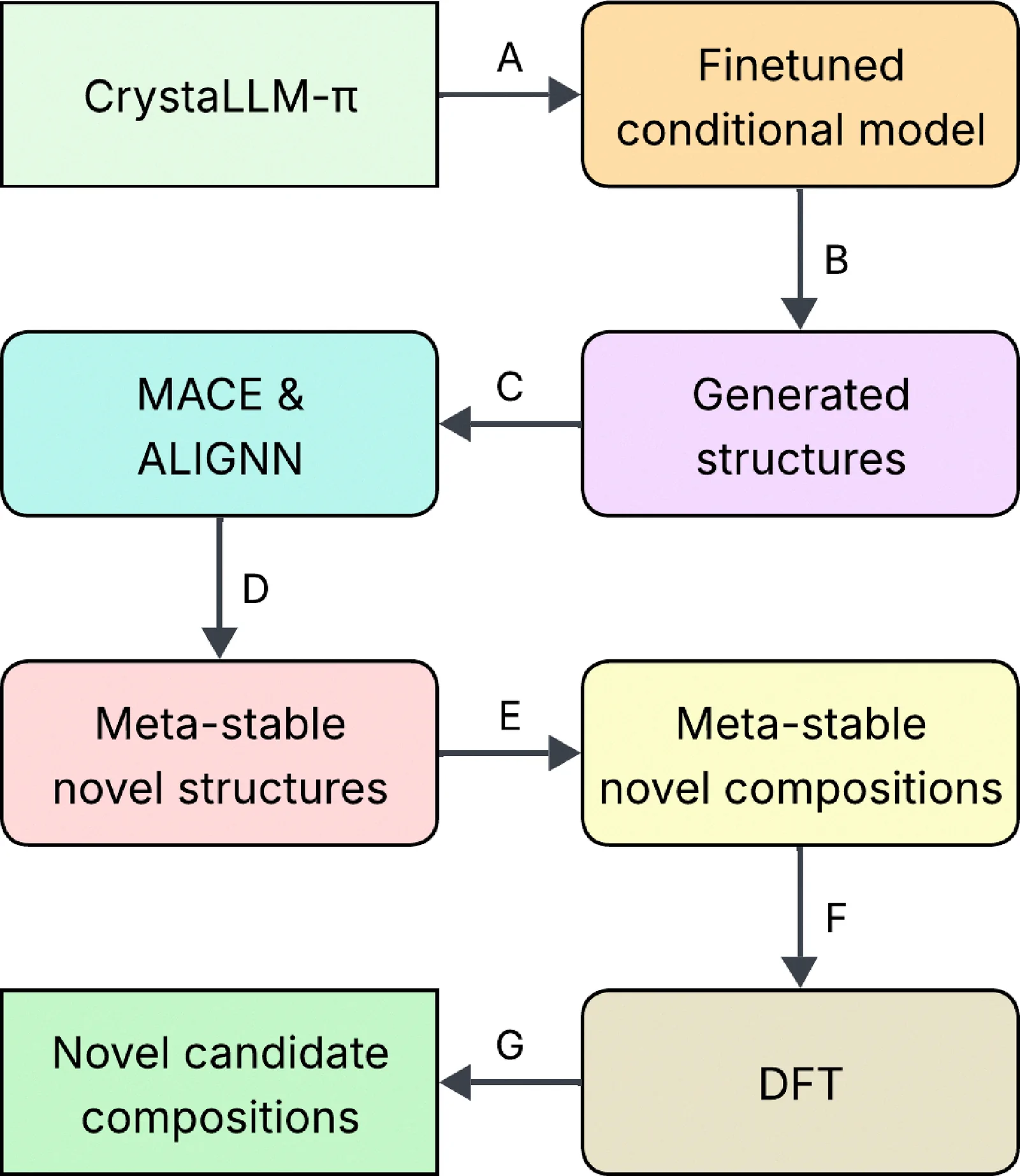
Full workflow for the conditional generative process.

Fig. 5 shows the frequency of each element in this set, together with the average *E*_hull_ of all structures containing that element. To avoid skewing colour intensities, the X-site elements were excluded, but as expected, all resolved to either carbon or nitrogen. Element counts were extracted from the reduced formula to provide stoichiometrically weighted prevalence across generations. Additionally, a stoichiometry-weighted frequency restricted to structures with *E*_hull_ < 0.05 eV per atom is reported to distinguish elements that dominate the most thermodynamically favourable half of the (*meta*) stable region.

Fig. 5 shows that Ti is the most prevalent M-site element, appearing with a stoichiometry-weighted frequency of 573 across 106 distinct structures and a mean *E*_hull_ of 0.042 eV per atom. Ti also appears frequently in the region corresponding to *E*_hull_ < 0.05 eV per atom, contributing a weighted frequency of 238 structures – the highest of any element. The prominence of Ti is expected, as Ti-based MAX phases were among the first discovered, widely studied, and synthesised, and their derivatives – particularly Ti_3_C_2_T_*x*_ – were also among the first MXenes reported.^2,36^ This indicates that the model is successfully recovering experimentally observed trends. Elemental prevalence across group 4 and 5 is also relatively consistent, with Sc (454), Hf (447), Zr (408), Ta (396), Nb (395) and V (341) all exhibiting substantial representation, however, the remaining M-site elements fall off sharply, where the next highest prevalence is observed in Mo which only notes a count of 189. Notably, Ta exhibits the lowest mean *E*_hull_ of any M-site element (0.032 eV per atom) despite ranking fifth by frequency, suggesting that Ta-containing MAX phases, while less frequently generated, are potentially more thermodynamically favourable when they do appear under this conditioning regime. Hf also shows a comparatively low mean *E*_hull_ (0.039 eV per atom) and the second highest count of *E*_hull_ < 0.05 eV per atom (189). Hf-containing MAX phases are however, less common in the literature,^37^ suggesting Hf-based compositions may be promising candidates for future study. Among the less prevalent M-site elements, W and Mn exhibit the highest mean *E*_hull_ values (0.058 and 0.068 eV per atom, respectively) and the lowest fractions of structures with *E*_hull_ < 0.05 eV per atom (30 and 6), indicating that while metastable W and Mn containing MAX phases exist within the generated set, they are less favoured by the imposed conditioning regime.

For the A-site, reduced prevalence is observed, though this is expected given stoichiometric weighting (A-site elements contribute one count per formula unit in M_*n*+1_AX_*n*_). Cd is the most frequent A-site element (109), followed closely by In (105), Ge (103), and Ga (102). Al, despite a lower overall frequency of 74, exhibits the lowest mean *E*_hull_ of any A-site element (0.031 eV per atom), followed by Ga (0.033 eV per atom). Both Al and Ga also show high representation for cases with *E*_hull_ < 0.05 eV per atom, noting stoichiometry-weighted frequencies of 38 and 54 respectively. The thermodynamic favourability of Al-containing MAX phases is consistent with experimental observations of Al as a common A-site element in both synthesised MAX phases and MXene-yielding precursors,^38^ while Ga, although less studied, features regularly in the literature,^4^ again indicating consistency between the model and well-established trends. Among other A-site elements, Ge achieves a mean *E*_hull_ of 0.039 eV per atom with the highest frequency of A-site elements possessing *E*_hull_ < 0.05 eV per atom (43), while Cd, despite its high overall prevalence, shows greater variance (*σ* = 0.050 eV per atom) and a median *E*_hull_ of 0.052 eV per atom, indicating a broader spread of output structures. At the less thermodynamically favourable end, Si and Pb exhibit mean *E*_hull_ values of 0.057 and 0.055 eV per atom, with commensurate lower counts of *E*_hull_ < 0.05 eV per atom (12 and 14, respectively).

Since all structures in this set already satisfy the 0.1 eV per atom metastability criterion, the relevant distinction lies not in whether elements pass a threshold but in the consistency of their stability. We are able to assess this by considering the standard deviation of *E*_hull_ values for each element. Elements such as Ta (*σ* = 0.047 eV per atom) and Sc (*σ* = 0.047 eV per atom) show relatively high variance, meaning that while their mean stability is favourable, individual compositions span a wider range. By contrast, Mn, despite having the highest mean *E*_hull_ among M-site elements, exhibits the lowest standard deviation (*σ* = 0.021 eV per atom), indicating that, CrystaLLM−π under the considered conditioning regime, can generate consistent structures within a tight thermodynamic range. These observations suggest that targeting high-frequency, low-variance elements – such as Ti, Hf, and Nb among M-sites, and Al and Ga among A-sites – may offer the most reliable generative route to thermodynamically favourable novel MAX phases.

Importantly, the fact that every M-site and A-site element in this set exhibits a mean *E*_hull_ well below 0.1 eV per atom confirms that viable candidates exist across the full range of conventional MAX phase chemistry, even if their relative abundance and thermodynamic favourability vary.

### 2.3. Novel composition generation and DFT validation

Across all non-compositional generations produced by the two conditional models under all condition vectors, a set of novel compositions were identified that appeared to correspond to metastable phases. Here, novelty is based on matching structures and compositions against the training data and is described in more detail in Section 4.7. These compositions were absent from both the base training set and the fine-tuning set. A list of these compositions, together with predicted bulk moduli and shear moduli, is provided in Table 1. All of these materials were confirmed to be dynamically stable by calculating their phonon density of states and confirming the absence of imaginary modes.

**Table 1:** Comparison of predicted (MACE) and DFT-calculated energies above hull, alongside mechanical properties (predicted by ALIGNN) and band gaps from DFT for the set of candidate MAX phases. Established novel metastable compositions as predicted by CrystaLLM−π and MACE

Formula	*E* ^MACE^_hull_ (eV per atom)	*E* ^DEFT^_hull_ (eV per atom)	*B* (GPa)	*G* (GPa)	Band gap (eV)
Mo_2_GeC_2_	0.00	0.02	236.47	93.85	0.00
VW_2_GeC_2_	0.10	0.23	243.41	84.31	0.00
ZrW_2_SiC_2_	0.10	0.11	246.42	86.74	0.00
Zr_3_GeC_2_	0.03	−0.04	167.07	72.30	0.00
Hf_2_ScInN_2_	0.03	0.05	174.75	81.06	0.00
HfZr_2_CdC_2_	0.00	−0.03	158.81	60.73	0.07
Sc_2_WPbC_2_	0.09	−0.01	101.79	70.25	0.00
Sc_2_TaInN_2_	0.10	0.16	136.03	66.65	0.00
ZrTi_2_SnC_2_	0.04	0.06	169.27	75.18	0.00
ScTi_2_CdC_2_	0.09	0.17	153.53	64.02	0.00

The ratio of shear to bulk moduli (G/B) generally falls between 0.4 and 0.6, indicative of ductility commonly associated with MAX phases.^38^ Additionally, structures listed in this table have, in some capacity, been included within the search space for MAX phases in prior literature. However, only one phase has been explicitly investigated to date: Zr_3_GeC_2_, which has been verified as theoretically stable through DFT calculations performed at the PBE level using the generalized gradient approximation (GGA).^39^ To the best of our knowledge, the remaining structures have not been explicitly investigated.

If we tighten our metrics further and define thermodynamic metastability as structures lying less than 0.050 eV per atom above the convex hull, we find that 5 out of the 10 candidates satisfy our criterion as seen in Table 1. Band gap calculations,at hybrid DFT level of theory, confirm that all candidates are metallic in character, another property commonly associated with MAX phases. In the case of the HfZr_2_CdC_2_ we might assume the small deviation from 0 eV is simply an artefact of calculation.

Considering the origin of these novel compositions, some emerge from regions of the condition vector space associated with a greater number of potential MXene derivatives and reduced A-site well curvature. As defined by the PKV_residual_ and/or PKV_prefix_ models, Hf_2_ScInN_2_ corresponds to a condition vector of (0.95, 0.40), Sc_2_PbWC_2_ to (0.95, 0.30), HfZr_2_CdC_2_ to (0.75, 0.30), TaMo_2_GeC_2_ to (1.00, 0.50), and Zr_3_GeC_2_ to (0.50, 0.75). Notably, TaMo_2_GeC_2_ and Zr_3_GeC_2_ were generated outside the primary region of condition vector space considered in this work. These arose from preliminary exploratory screening conducted early in the experimental workflow. Despite being outside our target conditioning space, these compositions were retained due to their compositional novelty relative to the training set.

Alternative discovery strategies for MAX phases include high-throughput substitutional screening workflows,^40^ where known MAX prototypes are systematically decorated with different chemical species and subsequently ranked according to machine-learned interatomic potentials or density functional theory calculations. Large-scale untargeted discovery efforts, such as the GNoME project,^8^ similarly identify stable materials from broad regions of inorganic chemical space before applying application-specific filters. These approaches have proven highly successful and are complementary to the strategy adopted here. The emphasis of the present work is not on exhaustive stability screening, but on directly steering generation toward regions of MAX phase space associated with desirable MXene-related characteristics. By incorporating conditioning variables linked to potential MXene derivative counts and A-site binding behaviour, the model is able to bias exploration toward compositions that may simultaneously exhibit thermodynamic viability and favourable exfoliation characteristics. In this sense, conditional generation provides a mechanism for targeted exploration rather than *post hoc* filtering of large candidate sets.

As a point of comparison, we examined the recently released GNoME stable-materials dataset and identified 102 MAX phases among approximately 381 000 reported stable materials. While this highlights the success of untargeted large-scale screening approaches, it also illustrates the relative sparsity of MAX phases within broadly generated materials datasets and motivates the use of domain-focused generative strategies when searching for MXene precursor materials.

While it reflects positively on model capability that feasible out-of-sample compositions can be generated within the targeted vector space, these calculations alone are insufficient to confirm that the resulting structures are able to be etched into MXenes. Considering the most common top-down synthesis route *via* HF acid etching, Björk *et al.*^41^ demonstrated that the chemical exfoliation process can be effectively understood by evaluating the free energy difference associated with removing A- and M-site elements, under the assumption that these species do not dissolve as a consequence of water splitting. This approach could therefore serve as a valuable subsequent screening step in future work to assess the etchability and exfoliation potential of generated MAX phases.

### 2.4. Low shot generation – borides

A total of 1670 MAB phase generations produced 619 valid boride CIFs, of which 28 were classified as novel and metastable under our defined metrics. These corresponded to multiple polymorphs of TaSiB, NbGaB, NbSiB, VSiB, CrSiB, and MoGaB. While these compositions have been previously reported, none have been documented in the crystal structures generated here to the best of our knowledge, thus qualifying as novel by our metrics. All of these materials were confirmed to be dynamically stable by calculating their phonon density of states and confirming the absence of imaginary modes.

DFT calculations on these structures revealed that 23 of the 28 candidates were metastable (*E*_hull_ ⪅ 0.051 eV per atom) under the defined level of DFT theory, and all exhibited a band gap metallic in character (zero band gap, calculated at hybrid DFT level of theory). While this demonstrates the model's capacity to recover physically reasonable structures in a low-shot regime, the diversity of the generated outputs remains limited: only four unique compositions out of the original six (MoGaB, NbSiB, TaSiB, and VSiB) satisfied the DFT-screening and all adopt a 1 : 1 : 1 MAB stoichiometry with the same *Cmcm* space group. In this regime, conditional and non-conditional generation rates closely track one another across the explored chemical space, with neither approach exhibiting a clear advantage. This convergence suggests that, under conditions of limited data availability, the model is unable to learn sufficiently strong or distinct structure–property relationships for conditional signals to meaningfully influence generation. Such behaviour likely reflects a combination of the aggressive learning rate employed during fine-tuning and the sparse representation of MAB-type structures in the training set. To more effectively integrate MAB phase generation into the broader workflow, future efforts should therefore focus on expanding and balancing the dataset to encompass a wider range of canonical MAB phases and stoichiometries, thereby alleviating the implicit constraints imposed by the low-shot regime.

### 2.5. Limitations

We note that both the training dataset and generated structures in this work are represented as fully ordered crystal structures. While this is consistent with standard crystallographic data and first-principles modelling approaches, experimentally realised MAX phases can exhibit varying degrees of crystallographic disorder, particularly within the transition-metal sublattice in multi-metal systems. Such disorder may include site mixing, partial occupancies, and local distortions that are not captured in ordered structural representations. Importantly, the MAX phase crystal structure is defined by its layered arrangement of M, A, and X sublattices; significant mixing between these sublattices—particularly between M and A sites—would disrupt this motif and likely preclude classification as a MAX phase. By contrast, disorder confined within a given sublattice (*e.g.*, partial mixing between transition metals on the M-site) is compatible with the MAX structural family and is observed experimentally. The structures generated here should therefore be interpreted as ordered prototypes corresponding to idealised representations of candidate phases. In practice, experimentally accessible materials derived from these prototypes may exhibit partial configurational disorder. Explicit treatment of disorder, for example through configurational sampling or large-scale atomistic modelling, represents an important direction for future work.

## 3. Conclusion

We have introduced a generative model, tailored to design new MAX materials. The model, based on CrystaLLM−π employs conditional autoregressive generation to propose new crystal structures biased towards having MAX structure and being amenable to exfoliation to MXene derivatives. To achieve this conditioning we developed two heuristics of MXene forming likelihood, one statistically derived from literature reports and one physically motivated based on bond strength. We demonstrate that these conditions can drive the underlying generative process to propose new materials that are physically more likely to have easy routes to exfoliation. We then further apply the model to propose new MAX materials and also specifically target the less explored MAB chemical space. We select some of the most stable novel compositions (as evaluated using the MACE machine learned interatomic potential) and test their stability and properties using DFT, identifying five new (*meta*)stable phases that to our knowledge have not been explored as promising MAX/MXene materials previously. We also use the model to expand the space of potential MAB materials – proposing 23 new structurally unique (*meta*)stable materials from DFT calculations.

## 4. Methods

### 4.1. General workflow

The complete investigative workflow is summarised in Fig. 6. In step A, we begin with a base model pre-trained on a diverse general corpus of approximately 4.25 million crystal structures,^42^ which is subsequently fine-tuned on a MAX-phase-specific dataset.^43^

In step B, this refined model is used for structural generation *via* a systematic sweep of the predefined condition vector, thereby exploring the target region of materials design space. At this stage, generation may proceed under either compositional or non-compositional prompting.

Step C comprises a multi-stage filtering process in which all generated structures are first validated for compliance with CIF semantic rules, internal structural consistency, and stoichiometric compliance with the expected (M,M′)_*n*+1_AX_*n*_ formula, where M, A and X are restricted to the elemental sets defined in Section 4.2, and structures also belong to one of the following space groups associated with MAX phases: *P*6_3_/*mmc*, *R*3̄*m*, *P*3̄*m*1, *P*6̄*m*2, *Cmcm*, and *P*6_3_*mc*. Structures that pass this screening are subsequently evaluated using the MACE and ALIGNN models to assess metastability (*E*_hull_ < 0.1 eV per atom) and predict relevant materials properties, enabling efficient elimination of unphysical or thermodynamically unstable candidates.

Within this valid MAX structure set, ∼25%, ∼26%, and ∼34% of valid MAX structures were classified as metastable (*E*_hull_ < 0.1 eV per atom) for the baseline, PKV_prefix_, and PKV_residual_ models respectively, prior to novelty filtering (step D). This disparity may be understood by considering the “softness” of the PKV_residual_ conditioning mechanism, which handles missing condition vector entries dynamically rather than through imputation, and therefore does not dilute the meaning of the condition vector across structural generations (see Section 4.4). Tightening the metastability requirement to *E*_hull_ < 0.05 eV per atom narrows this gap to ∼13%, ∼15%, and ∼16% respectively, suggesting that the excess metastable fraction under PKV_residual_ conditioning is concentrated toward the upper end of the metastability window rather than reflecting a uniformly higher-quality generation distribution. For context, at the original *E*_hull_ < 0.1 eV per atom threshold, the corresponding novel-and-metastable generation rates reported in Section 4.6.1 are lower still – 3.25% (baseline), 2.32% (PKV_prefix_), and 2.10% (PKV_residual_) – since novelty is a further restrictive filter applied downstream at step D.

In step D, candidates that are both physically plausible and thermodynamically reasonable are screened for structural novelty against both the base dataset and the MAX-phase fine-tuning set, ensuring that only stable and structurally unique outputs are retained. Structural novelty is assessed through symmetry-based screening, in which candidates are considered distinct only if they differ beyond defined tolerances: a 0.1 Å criterion for symmetry-equivalent positions, a 0.3 Å site displacement tolerance, a 20% variation in lattice parameters, and a 5° angular tolerance.

These *meta*-stable, structurally novel candidates are subsequently filtered based on chemical composition, with novelty again assessed relative to the full training and fine-tuning corpus (step E).

In step F, the remaining *meta*-stable and compositionally novel structures are subjected to density functional theory (DFT) calculations, providing a more stringent evaluation of thermodynamic stability. Finally, in step G, the candidates are filtered according to their DFT-derived energies and cross-referenced with their originating regions of condition vector space, yielding a final set of compositionally novel structures that are likely to exhibit desirable MAX-phase characteristics.

### 4.2. Data collection

This study makes use of a dataset of 8591 double transition-metal MAX phase structures generated using DFT at the PBE level.^43^ These structures underwent ionic relaxations with a stopping criterion of 10^−5^ eV and cell relaxations with a stopping criterion of 10^−6^ eV, with convergence ensured by employing a plane-wave cutoff energy of 550 eV and a *k*-point grid of 18 × 18 × 3. Within the domain of double transition-metal MAX phases, this database has constituted the largest collection of its kind since 2023.

**Fig. 7:**
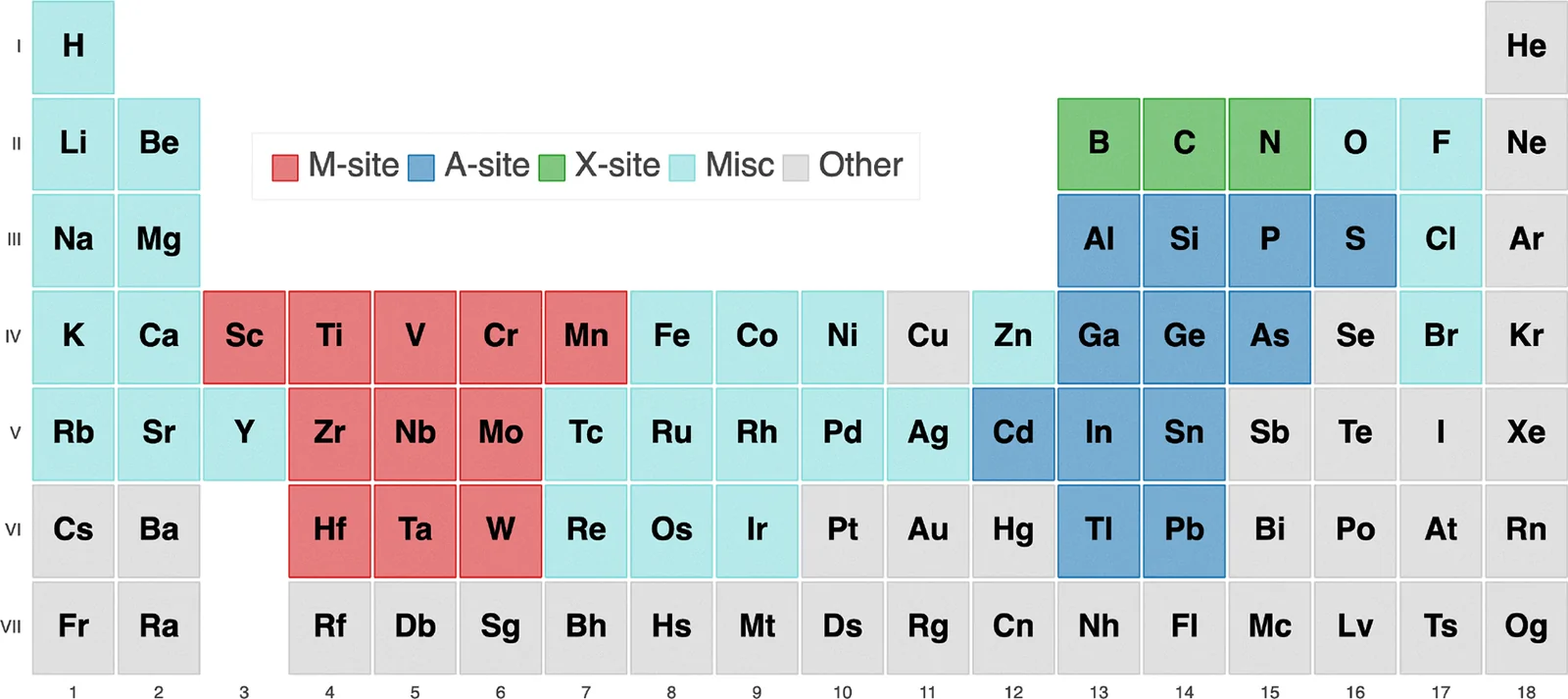
The elemental space of the modified fine-tuning dataset including miscellaneous elements that appear in the MAX/MAB-like set.

To complement this, an additional set of 166 compositionally unique MAB and MAX-like structures was obtained from the materials project and NOMAD databases. These were filtered based on theoretical stability, exclusion of rare-earth elements, and assignment to one of the following space groups: *P*6_3_/*mmc*, *P*6_3_*mc*, *P*6_3_/*mcm*, *R*3̄*m*, *Cmcm*, *Pnma*, *Cmmm*, *P*6̄*m*2, *P*3̄*m*1, and *P*6_3_. This step enriched the dataset with additional chemical and structural diversity, particularly for boride compounds (104 in total, all classified as metastable by the materials project, NOMAD, and the stability metrics employed in this study) as well as MAX-like compounds. This was necessary because the original double transition-metal MAX phase dataset contained only carbides and nitrides, and therefore did not capture or even vaguely reference the distinct chemistry of MAB phases (M_2_AB_2_, M_3_AB_4_, M_4_AB_6_, M_4_AB_4_, M_2_A_2_B_2_).^4^ Overall, 6179 species in this dataset emerged as structurally unique under our metrics and were therefore passed forward to be used in model fine-tuning. The elemental space of the fine-tuning dataset is illustrated in Fig. 7.

An additional dataset comprising 23 857 MXene phases, also generated using DFT at the PBE level (the aNANt functional materials database^44^), was also considered. This dataset was specifically used to define the MXene derivative count dimension of the condition vector.

### 4.3. Thermodynamic stability evaluation

Total energies were calculated using the MACE-MP (message passing atomic cluster expansion) calculator,^5^ employing its default model pre-trained on materials project structure relaxation trajectories. To assess phase stability, formation energies of known materials from the materials project were used to construct a convex hull.

The convex hull provides a straightforward means of determining thermodynamic stability within a chemical system. It is constructed by plotting the formation energies of all known or theoretically predicted compounds against their compositions and connecting the lowest-energy points to form a “hull”, which represents the set of compositions stable against decomposition into other phases. Compounds that lie on the hull are thermodynamically stable, whereas those positioned above it are unstable and tend to decompose into combinations of phases residing on the hull.^45^

From this framework, the *E*_hull_ metric was defined as the energy above the convex hull for each structure, calculated by comparing its formation energy against the most thermodynamically stable competing phases with the same composition and stoichiometry. All competing phase energies were computed using MACE-MP. Structures with *E*_hull_ < 0.1 eV per atom were classified as *meta*-stable.^46^

### 4.4. Property key value (PKV) conditioning

Two conditioning methods were considered in this study. The first, denoted PKV_prefix_, incorporates a learnable embedding of the condition vector directly into the attention mechanism, representing a stronger form of conditioning. The second, PKV_residual_, provides a softer alternative in which two attention scores, one attending to the sequence and one to the conditioning information, are computed in parallel, where the next token prediction is obtained as a weighted sum of these contributions. This formulation allows PKV_residual_ to naturally handle missing values in the condition vector.^31^

Missing values were treated using two distinct strategies. The first model, PKV_residual_, is inherently capable of handling missing entries in the condition vector by dynamically adjusting how information is injected into the model's attention mechanism. The second model, property key value (PKV_prefix_), required explicit imputation, which was carried out by substituting the normalised mean value for each missing instance.

Owing to the structural and compositional differences between MAB/MAB-like and canonical MAX phases, all condition vectors were imputed for boride structures during training. Although sub-optimal for low-shot generation, this approach enables uniform algorithmic treatment of all training structures, ensuring consistency across this experimental framework. Independent MAB phase conditioning may therefore, be a valuable direction for future investigation.

### 4.5. Fine-tuning and hyperparameter optimisation

The fine-tuning dataset was divided into training, validation, and test sets in an 80 : 10 : 10 ratio. Borides were withheld from the test set to maximize their representation in the training and validation sets under a low-shot regime. The test set was used solely to verify outputs prior to exploratory MAX phase generations and is not considered further in this work. Hyperparameter tuning was conducted over a restricted search space by sweeping a grid of 20 plausible configurations, varying the learning rate, prefix token, and hidden condition size. The optimal model was selected on the basis of the lowest validation loss achieved.

Three models were subsequently fine-tuned: the PKV_prefix_ model, the PKV_residual_ model, and a baseline model without a condition vector. Where applicable, all models were trained under identical settings to ensure a fair and consistent comparison.

### 4.6. Structure generation

Structure generations were carried out using both compositional (MAB phases) and non-compositional prompting. For the conditioning models, a series of condition vectors was additionally swept across both prompting modes.

#### 4.6.1. Non-compositional prompting

Multiple investigations were carried out under this regime. First, 1000 structures were generated for each model type at a fixed MXene derivative count condition of 0.95, while sweeping the A-site well curvature condition from 0.00 to 1.00 in increments of 0.10. Raw well curvature values were then calculated for each generated structure to assess the influence of the A-site well curvature condition on generation outcomes. In this instance, structures were filtered only for validity meaning that energy ranges and novelty were not considered. Significance was again assessed at the 5% level using a two-sided Pearson correlation test, with the null hypothesis that there is no correlation between the A-site well curvature condition and the measured curvature of the generated structures.

Non-compositional prompts were swept across a narrower range: 0.75 to 0.95 (inclusive, step size 0.10) for the MXene derivative dimension, and 0.0 to 0.4 (inclusive, step size 0.10) for the A-site well curvature dimension. This produced 15 distinct condition vectors. These ranges were chosen pragmatically, as they correspond to regions where many potential MXene derivative candidates are defined and where weak A-site binding is suggested by low curvature values. Structures from this sweep were then filtered on the basis of novelty, validity, and metastability (*E*_hull_ < 0.1 eV per atom). See Table 2 for a qualitative interpretation of what these condition vectors mean.

**Table 2:** Examples of conditioning vectors and their qualitative interpretation

Condition vector	Qualitative interpretation
(0.75, 0.00)	Lower MXene-derivative score within the swept region and weakest A-site binding (lowest well curvature).
(0.85, 0.20)	Intermediate MXene-derivative score with moderate A-site well curvature in the explored range.
(0.95, 0.40)	Highest MXene-derivative score within the swept region and strongest A-site binding (highest well curvature).
(0.95, 0.00)	Highest MXene-derivative score within the swept region combined with weakest A-site binding (lowest well curvature).

In this setup, 19 800 structures were generated for each conditional model type, yielding 1320 structures per condition vector. For comparison, the baseline model generated 19 800 structures without conditioning.

To assess the significance of novel stable structure generation rates, in both cases a Fisher's exact test was performed at the 5% significance level, comparing the performance of individual condition vectors for both model types against the baseline model. The null hypothesis was that the use of a specific condition vector does not alter the rate of novel stable structure generation relative to the baseline model.

#### 4.6.2. MAB phases – low shot generations

Due to reduced prevalence of MAB phases in the fine-tuning set, boride structures accounted for only 104 of 6179 entries (1.68%), providing a suitable case study for assessing the low-shot generation capabilities of CrystaLLM−π in this context. Faithful MAB phases represent an under-explored subclass of MAX-type structures, noted for adopting distinct structural motifs and stoichiometries.^4^ These phases can be etched into MBenes analogously to MXenes and are emerging as promising alternatives for applications in batteries, supercapacitors, and catalysis.^47^

To systematically investigate boron-containing compositional space within this low-data regime, a series of 167 unique compositional prompts was generated using a predefined set of elements, following the stoichiometric rules of MAB phases. Prompt compositions were validated using SMACT^48^ to ensure charge neutrality and electronegativity balance. For each of the 167 prompts, 10 structures were generated, yielding a total of 1670 generations. The generation process followed a prompting methodology where condition vectors ranging from 0.00 to 1.00 were swept in increments of 0.25 for each dimension, resulting in 25 distinct condition vector sets. This conditioning strategy was applied uniformly across both PKV_prefix_ and PKV_residual_ model variants, enabling direct comparison of their respective performance in the boride chemical space.

### 4.7. Structure matching and novelty evaluation

Structural novelty was assessed using the pymatgen StructureMatcher tool.^49^ Matching was performed with the following thresholds: 0.1 Å for symmetry equivalence, 0.3 Å for site equivalence, 20% variation in lattice parameters, and a 5° variation in lattice angles. Structures exceeding all thresholds were designated as structurally novel.

Compositional novelty was determined by direct comparison of pymatgen reduced formula composition objects from the generated set against those present in the full training dataset.

### 4.8. DFT calculations

DFT calculations were performed using VASP with a plane wave cutoff of 520 eV. Structural relaxation used the PBE functional with the projector augmented wave (PAW) method. Electronic convergence was set to 10^−6^ eV and ionic convergence to 0.05 eV Å^−1^. Structural relaxations were carried out in three regimes: (i) relaxation of atomic positions (ISIF = 2), (ii) relaxation of both atomic positions and lattice vectors (ISIF = 3), and (iii) short refinement runs. The conjugate-gradient algorithm was used with Gaussian smearing (*σ* = 0.05 eV, ISMEAR = 0). Final static total-energy calculations were performed using the tetrahedron method with Blöchl corrections (ISMEAR = −5) and no relaxation of ions (IBRION = −1). *k*-Point meshes were automatically generated using Pymatgen's MPRelaxSet and MPStaticSet to ensure compatibility with the materials project database. The maximum relaxation cycles were capped at 20.

Bandgaps were evaluted using the HSE06 functional^50,51^ through a single point calculation on the relaxed structures. To test their dynamic stability, we perform finite displacement calculations using phonopy^52^ with a 3 × 3 × 2 supercell for the *P*6_3_/*mmc* and a 4 × 4 × 4 supercell for the *Cmcm* structures using the PBE functional. A higher ENCUT value of 580 eV was used for the phonopy calculations. A Monkhorst–Pack *k*-grid was used with a min *k*-density of 5 *k*-points per Å^−1^.

## Author contributions

KTB and JS conceptualised the project and wrote the initial manuscript. CB provided technical supervision and reviewed the manuscript. EG and MD provided guidance on chemical systems and contributed to conceptualisation and development of the project and manuscript.

## Conflicts of interest

There are no conflicts to declare.

## Data Availability

All data and code needed to recreate the results presented in this paper are available from https://github.com/j-swaine/Conditional-Generation-of-MAX-Phases (code) and https://huggingface.co/datasets/Jamie1701/conditional-generative-models-max-phase (data). Phonon band structures and outputs from hybrid DFT calculations of new metastable materials are available at https://doi.org/10.5281/zenodo.21457147.
